# Pain for pain: the benefits and challenges of BDSM participation for people with chronic pain – An exploratory study

**DOI:** 10.1080/19419899.2025.2507699

**Published:** 2025-05-21

**Authors:** Reni Forer, Bryce Westlake

**Affiliations:** a University of Michigan Medical School, Ann Arbor, MI, USA; b Department of Justice Studies, San Jose State University, San Jose, CA, USA

**Keywords:** BDSM, chronic pain, mental health, quality of life, edge play

## Abstract

Research is beginning to find that many BDSM (bondage/discipline, domination/submission, sadism/masochism) practitioners experience benefits beyond sexual pleasure. Additionally, given the relationship between pain and pleasure common in BDSM, there may be certain unanticipated benefits for practitioners reporting chronic pain (PRCP). We conducted an online survey of an international convenience sample of BDSM practitioners (N = 525). Participants were asked about self-perceived benefits and challenges related to participating in BDSM with CP. Using chi-square, independent sample t-test, logistic regression, and linear-by-linear association, responses were combined with demographics, participation timelines, co-participants, motivations, and frequency of activities to identify differences between PRCP and not reporting CP. PRCP do not appear to initially seek out BDSM for pain management but report both physical and mental benefits and identify mental health as a motivation for continued participation. Many PRCP also report at least some short-term pain relief resulting from BDSM participation. Importantly, they are more likely to engage in higher intense sensation activities (e.g. edge-play) more frequently. This study provides a foundation for further investigation into the nuanced relationship between CP and BDSM participation, especially in relation to the intensity and type of activities engaged in.

## Introduction

BDSM is a blended acronym encompassing bondage/discipline, domination/submission, and sadism/masochism and is increasingly being recognised as a common variant along the spectrum of human sexual preferences and practices (e.g. Strizzi et al., 2022). While BDSM can have a positive impact on sexual satisfaction (Botta et al., 2019; Joyal & Carpentier, 2017), research has begun to demonstrate that there can be non-sexual reasons for engaging in BDSM (Sagarin et al., 2015; Westlake & Mahan, 2023). Participation can also benefit other aspects of one’s life, including trauma-processing (Lehman et al., 2024; Thomas, 2020), decreased psychological distress and rejection sensitivity (Hébert & Weaver, 2015; Sprott & Williams, 2019), and higher subjective well-being (Turley, 2024; Wismeijer & van Assen, 2013). Given these mental health-related benefits, as well as the reported experience of certain people finding acute pain pleasurable during BDSM activities (Dunkley et al., 2020; Pollok et al., 2010), another possible domain where BDSM participation may have a positive impact is in the management of chronic pain (CP).

### Defining and treating chronic pain

Pain is defined by the International Association for the Study of Pain as an unpleasant sensory and emotional experience associated with, or resembling, actual or potential tissue damage (Raja et al., 2020). Meanwhile, CP is defined as any pain that lasts for greater than three to six months, and may or may not have a clear aetiology, such as an injury or disease (Dydyk & Conermann, 2024). CP encompasses multiple conditions and experiences, which can manifest through various types of pain, alone or in combination, that affect work-up and treatment (Cohen et al., 2021). These conditions can present at any time from childhood and adolescence to older age (King et al., 2011; Larsson et al., 2017).

CP is estimated to affect more than 30% of people worldwide (Cohen et al., 2021), with European residents reporting the highest prevalence (34%) and Western Pacific residents reporting the lowest at 21% (Zimmer et al., 2022). CP disproportionately affects women (Tsang et al., 2008), older adults (Tsang et al., 2008), certain racial/ethnic groups (e.g. Native Americans) (Overstreet et al., 2023; Zajacova et al., 2022), and people of lower socioeconomic status (Prego-Domínguez et al., 2021) and lower education level (Dahlhamer et al., 2018). Finally, experiencing CP increases the risk of developing symptoms associated with various psychiatric diagnoses, such as depression and anxiety-spectrum disorders (Tsang et al., 2008; Vadivelu et al., 2017).

As there is no ‘one size fits all’ treatment for CP, people living with CP (PLWCP) are increasingly turning to non-pharmacological treatments to complement pharmacological options for pain management (Dydyk & Conermann, 2024; Holtrop et al., 2019). The efficacy of these alternative non-pharmacological approaches is mixed, with one comprehensive review finding that 59–90% of patients found them to be beneficial (Urits et al., 2021). Paradoxically, some of these approaches involve a high likelihood of intense sensory experiences, which can include temporary pain, discomfort, and/or stress. Examples include acupuncture (Giovanardi et al., 2023), certain types of physical exercise (Fernández-Rodríguez et al., 2022), and transcutaneous electronic nerve stimulation (Johnson et al., 2022). One rationale for this approach is the phenomenon of ‘pain offset relief’, where removal of a painful stimulus that is causing an unpleasant physical sensation does not simply return individuals to their pre-stimulus or neutral state (e.g. in CP) but rather results in a short period of relief or euphoria (Franklin, 2014). This phenomenon has been linked to the activation of certain brain circuits and neurotransmitters, including dopamine and opioid transmission (Porreca & Navratilova, 2017). Another rationale for some of these treatments, namely those that involve peripheral nerve stimulation such as acupuncture and TENS (Johnson et al., 2022; Ong Sio et al., 2023) is the gate theory. This theory suggests that applying non-painful stimuli to non-nociceptive neurons causes activation of inhibitory interneurons and therefore inhibition of certain nociceptive neurons that would otherwise transmit pain signals to the central cortex (Melzack & Wall, 1965).

### The relationship between BDSM and chronic pain

BDSM includes a variety of activities typically centred around exchange of control, power, and/or intense sensation between consensual partners (Dunkley et al., 2020). It has been suggested that some practitioners engage in BDSM *because* of pain and the sense of pleasure/euphoria they receive from it (Dunkley et al., 2020; Silva, 2015). For example, among people who engage in pain-receiving (e.g. masochistic) behaviour, it has been demonstrated that pain was experienced as pleasurable and rewarding during BDSM activities and remained pleasurable, although not rewarding, in everyday life (Defrin et al., 2015). The proposed biological mechanism behind this sense of pleasure from pain is the release of certain stress hormones (Larva & Rantala, 2024; Wuyts et al., 2020) and changes to dopamine, cortisol, endogenous opioid, and endogenous endocannabinoid levels (Dunkley et al., 2020). Given the overlap in neurotransmitters and brain circuitry involved, BDSM could unknowingly result in pain offset relief for participants with CP, providing temporary relief from, or reduction in, CP. As PLWCP cite reducing the intensity of pain as their priority for management (Goudman et al., 2021), these unanticipated benefits may serve as a motivating factor for continued participation in BDSM, and in specific activities that are more likely to facilitate an intense sensory experience and elicit a pleasurable sensation.

Research exploring the relationship between CP and BDSM has been extremely limited. In their graduate thesis, Jobson (2020) used a feminist and cripistemologic approach to discuss how BDSM practices allow people to challenge CP through expression, recognition, and discussion. They also considered whether those with CP could use participation in BDSM to change their relationship with pain. Sheppard (2018) conducted a series of interviews with eight disabled PLWCP who participated in pain-related BDSM activities. Interviewees noted that BDSM participation helped them to improve their self-esteem, reclaim their bodies, reframe thoughts around pain, distract themselves from pain, and even experience pain relief. This work is discussed further in an exploration of the use of BDSM to control pain (Sheppard, 2019). Finally, K. Brown and Iverson (2023) presented a case study of a patient with endometriosis who used BDSM to control and displace her CP. Combined, these suggest that there may be pain management benefits obtained from engaging in BDSM; however, the isolation of these investigations to a handful of participants necessitates more thorough investigation.

In the current study, we aimed to provide a more robust exploration of CP and BDSM participation. To accomplish this, we focused on BDSM practitioners reporting experiences of CP (PRCP) and their perceived benefits of BDSM for pain management, the challenges their CP created in participating in BDSM, and how their engagement in BDSM (entry into BDSM, motivations, co-participations, and frequency of certain activities) may differ from BDSM practitioners without CP.

## Materials & methods

### Participants

Self-described BDSM practitioners were recruited to participate in an online survey via social media, including FetLife (a Facebook-style BDSM discussion website), Reddit, and Facebook; university kink and/or sexuality organisations; and non-university kink and/or sexuality organisations (e.g. Kinsey Institute). Participants were offered a lottery incentive for their time, separate from their responses to retain anonymity. The survey covered nine broad topics related to motivations, benefits, and experiences to BDSM participation (see Westlake & Mahan, 2023 for in-depth survey description). Overall, 979 people attempted (i.e. completed at least 5%) the anonymous Qualtrics survey, with 595 (61%) completing the full survey. Among the topics covered was an optional section on mental health. Within this section were a series of questions pertaining to CP. The current study consists of the 525 participants who responded to the optional question ‘Do you have chronic pain?’ Of these, 305 (58.1%) said ‘no’, 201 (38.3%) said ‘yes’, and 19 (3.6%) said ‘yes, but I want to skip the questions about chronic pain’.

General and BDSM-related demographics of respondents to the CP question are provided in Tables 1 and 2 respectively. Overall, participants came from 34 different countries, with the majority being from the US (62.1%), Canada (14.7%), and the United Kingdom (7.7%). They contained diverse relationship statuses and age ranges, with Millennials (born between 1981 and 1996) accounting for 45.5% of respondents. Participants predominantly identified as assigned female at birth (62.4%), women (50.7%), heterosexual (38.0%) or bisexual (31.6%), White (84.4%), highly educated (63.2% with a bachelor’s or graduate degree), and employed (75.0%). Nearly half (48.3%) identified only with submissive roles, while 30.1% identified with both dominant and submissive roles (e.g. switch). Finally, nearly 20% of participants identified as masochistic (i.e. deriving pleasure from pain).

### Materials

The survey development was informed by interviews with community leaders and underwent external review by BDSM community and non-BDSM-community members and organisations. The final version of the survey was approved by the San Jose State University Institutional Review Board (21–163). All 525 respondents were asked a series of questions about their participation in BDSM. This included, first, recalling the age at which they ‘first learned about BDSM’, ‘first became interested in BDSM’, ‘first participated privately in BDSM’, and ‘first participated publicly in BDSM’. Years of BDSM experience was determined by subtracting the age at which they first participated in BDSM from their current age. Second, their source(s) of introduction to BDSM: ‘self’, ‘friend’, ‘partner’, ‘family member’, ‘other’, and ‘don’t know’. Third, with whom they participate in BDSM, including one or more of: ‘sex and romantic partner’, ‘non-sex partner’, ‘sex-only partner’, ‘BDSM-only partner’, ‘friends’, ‘strangers (i.e. pick-up play)’, ‘alone’, ‘with a professional’, ‘online’, and ‘other’. Fourth, their reason/purpose for BDSM participation. This open-ended question was coded into six themes and 22 sub-themes (for methodology details see Westlake & Mahan, 2023). One sub-theme that emerged was ‘mental’, which consisted of responses such as ‘therapeutic’ or ‘coping’. Fifth, their frequency of participation in ‘rope’, ‘impact’, ‘fire’, ‘water’, ‘blood’, and ‘cutting’ activities using a five-point Likert scale from ‘never’ to ‘regularly’.

The 201 participants who responded ‘yes’ to the optional question, ‘Do you have chronic pain?’ were administered 16 questions pertaining to BDSM participation, each measured on a five-point Likert scale ranging from ‘not at all’ to ‘regularly’. The first nine questions asked about how BDSM positively impacted CP. Five of these asked about mental benefits: ‘empowering with consensual pain’, ‘improving ability to speak about pain’, ‘managing pain through negotiation’, ‘engaging emotionally with pain’, and ‘re-framing limitations through exploring creative alternatives’. The remaining four asked about the physical benefits: ‘masking non-consensual pain’, ‘providing pain relief’, ‘creating an endorphin rush’, and ‘strengthening pain tolerance’. ‘Endorphin rush’ was used due to endorphins typically being the hormones attributed to short-lived euphoric states both historically and in popular vernacular (Siebers et al., 2023). The remaining seven questions asked about how CP negatively impacted BDSM participation. Five of these were related to mental drawbacks: ‘feeling discouraged’, ‘feeling excluded’, ‘difficulties finding partners’, ‘challenges in getting needs met’, and ‘challenges getting wants met’. Two pertained to physical hindrances: ‘feeling discomfort’ and ‘additional pain’.

### Analytical procedure

To investigate potential positive and negative relationships between CP and BDSM participation, four research questions were explored through a series of univariate and bivariate analyses. First, what physical and mental benefits and challenges are endorsed by BDSM PRCP? This was explored through univariate analyses of the 16 self-reported benefits and challenges questions.

Second, are certain demographics of PRCP overrepresented in BDSM communities, when compared to their prevalence in the general population, and are certain BDSM roles (e.g. submissives) more likely to be occupied by PRCP? These were examined using a series of chi-square analyses comparing participants reporting and not reporting CP. For 2 × 2 chi-squares (e.g. sex assigned at birth), phi was reported for effect size. For larger analyses (e.g. 2 × 4), Cramer’s V was reported, as it corrects for the number of rows and columns.

Third, do BDSM timelines differ for PRCP when compared to those that do not report CP? This was explored through four independent sample t-tests conducted on the age participants first learned about, became interested in, and subsequently participated privately and then publicly, in BDSM. For each, a Levene’s test was used to determine equality of variance and Cohen’s d was calculated to determine effect sizes.

Fourth, does duration, co-participants, purpose, and/or frequency of participation in BDSM activities differ between those who do and do not report experiencing CP? This was explored through three separate analyses. First, through an independent sample t-test, we sought to identify whether more years of BDSM experience was associated with lower rates of CP being reported, potentially pointing to CP contributing to people ceasing their participation in BDSM. Second, through a logistic regression analysis, we examined how engagement in BDSM with others (both initially and continually) and the reason for continued participation in BDSM may predict practitioners reporting CP. Three variables were included in this analysis—three common sources of introduction (self, partner, friend), four types of co-participants (alone, sex/romantic partner, strangers, friends), and citing ‘mental health’ as a purpose/reason for participating in BDSM. Model fit was determined using the Hosmer and Lemeshow Test. Third, through six linear-by-linear associations (also known as the Mantel–Haenszel test), we investigated whether CP may be associated with engaging in more extreme and/or pain-related activities more frequently. Linear-by-linear associations were chosen because of the ordinal nature of the frequency variables and therefore odds are more appropriate than probability. Cramer’s V was calculated to determine effect size.

## Results

### The benefits and challenges to participating in BDSM while experiencing CP

**Figure 1.**
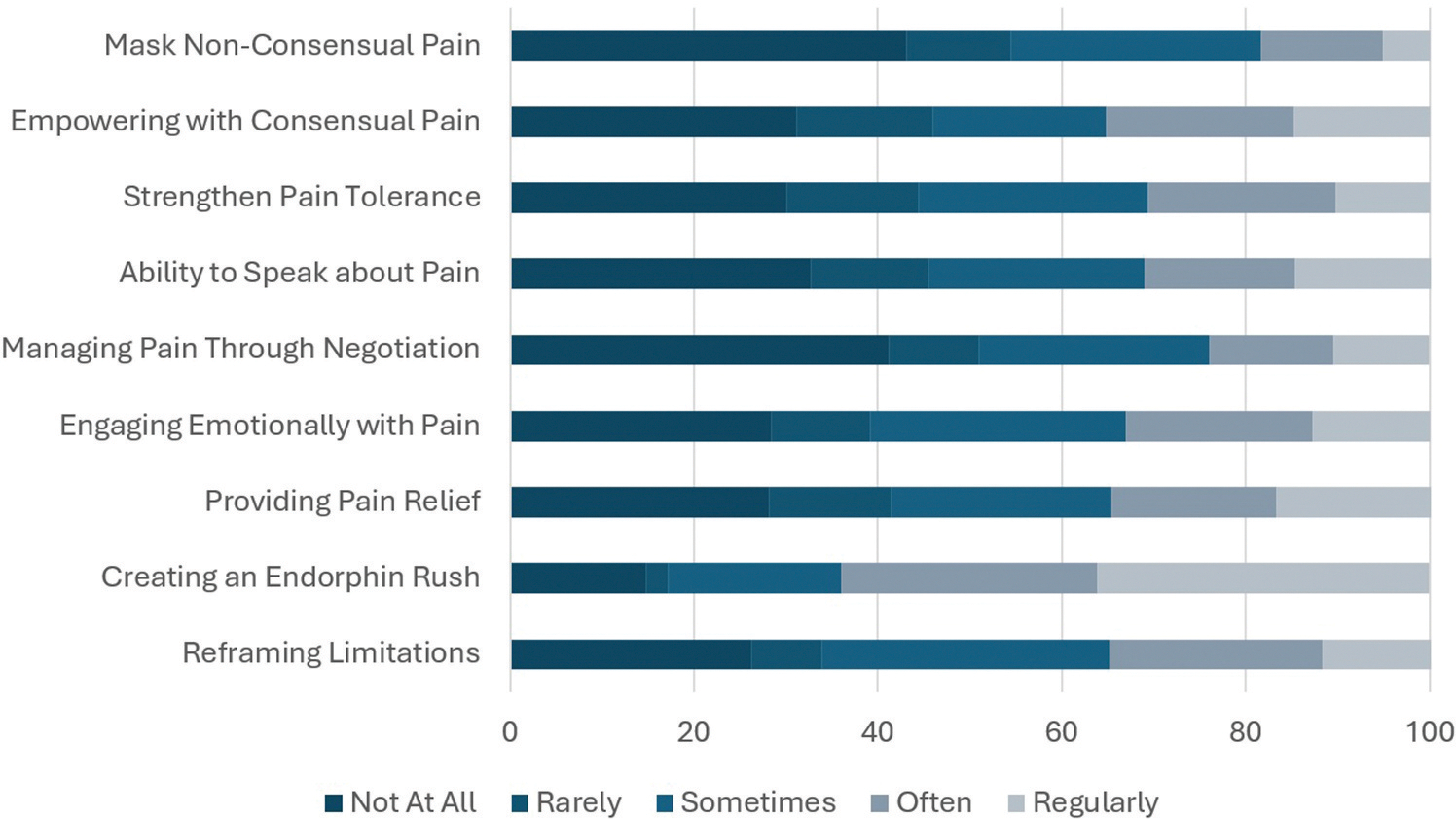
Physical and mental benefits of participation in BDSM for practitioners reporting chronic pain.

BDSM PRCP experienced multiple physical and mental benefits ‘often’ or ‘regularly’ (Figure 1). The most common benefit was an endorphin rush (63.9%), while more than 30% reported empowerment (35.2%), reframing of limitations (34.9%), pain relief (34.7%), engaging emotionally with pain (33.0%), speaking about pain (31.1%), and strengthening pain tolerance (30.6%) as benefits. The least common benefit was masking non-consensual pain (18.4%). In contrast, some PRCP did not appear to receive CP-related benefits from BDSM participation. More than 30% reported ‘not at all’ for managing pain through negotiation (41.1%), speaking about pain experienced (32.7%), empowerment (31.1%), and strengthening pain tolerance (30.1%).

**Figure 2.**
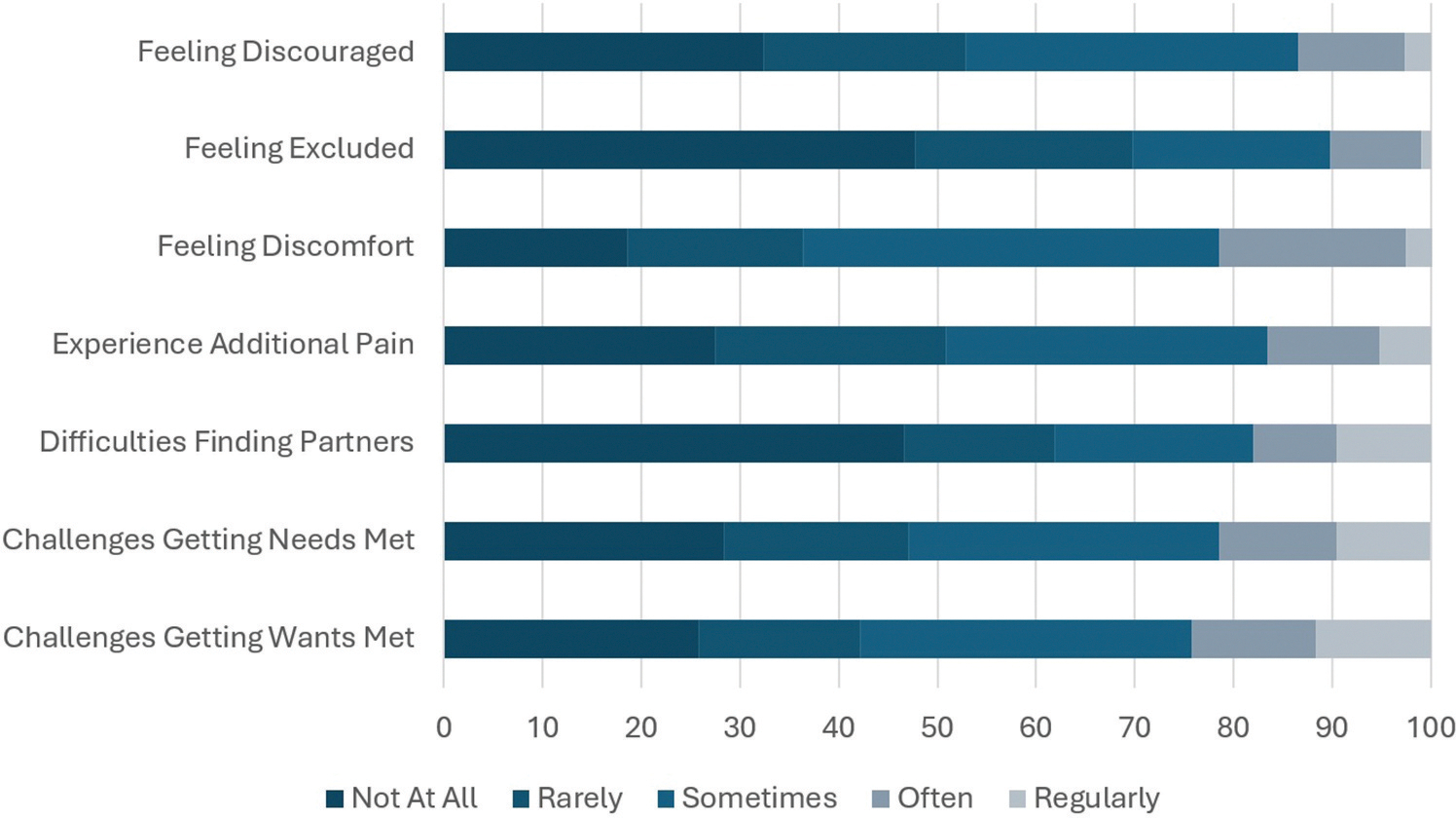
Physical and mental challenges of participation in BDSM for practitioners reporting chronic pain.

There were also challenges to BDSM participation experienced by PRCP. At least 20% reported ‘often’ or ‘regularly’ to three hindrances (Figure 2). These were feeling discomfort (21.6%) and getting wants (24.2%) or needs (21.6%) met. Conversely, almost half stated that they never (i.e. ‘not at all’) experienced feeling excluded (47.7%) or had difficulties finding co-participants (46.6%). CP also did not appear to commonly lead to feelings of discouragement, with 32.3% reporting ‘not at all’.

### Comparing demographics and timelines of BDSM practitioners reporting and not reporting CP

Demographic differences between those reporting and not reporting CP are compared in Table 1. Those assigned female at birth (67.2% PRCP versus 53.9% without CP), along with those who identified as women (56.2% vs 46.7%), were more likely to report experiencing CP. Effect sizes for both sex (0.13) and gender (0.14) were small. Likewise, there was a small effect for the country of residence (0.16) with people in the US being more likely to report experiencing CP (68.8% vs 57.2%), while those in the United Kingdom (5.0% vs 9.7%) and other parts of the world (excluding Canada) (10.6% vs 20.1%) were less likely. Finally, there was a small effect for education level (0.15) and occupation status (0.13). Those who were employed (73.0% vs 83.0%) and those with graduate degrees (19.3% vs 31.6%) were less likely to report experiencing CP, while students (10.0% vs 5.2%) and those with only some college (33.9% vs 24.0%) were more likely.

Examining specific BDSM roles (Table 2), PRCP were no more likely to occupy dominant (21.4% vs 21.8%) or submissive (48.3% vs 48.1%) roles than those without CP. Likewise, both were equally likely to identify with the role of a sadist (12.9% vs 15.9%). While there was no significant difference found between those who identified as masochists (22.6% vs 15.9%), this analysis approached significance (X^2^ (1, *N* = 519) = 3.715, *p* = 0.054); however, the effect size (0.09) was small to negligible.

Table 3 shows that there was also no significant difference in the average age at which those reporting and not reporting CP first learned about (t(511) = 0.27, *p* = 0.784), became interested in (t(517) = 0.38, *p* = 0.705), participated privately in (t(515) = 0.71, *p* = 0.476), or participated publicly in (t(251.88) = 0.71, *p* = 0.477) BDSM. Effect sizes were negligible, ranging from 0.03 to 0.08. Although those reporting CP had, on average, slightly more years of BDSM experience (16.77 years vs 15.04 years), this difference was not significant (t (512) = 1.56, *p* = 0.120) and the effect size was negligible (0.14).

### Differences in source of introduction, purpose for participation, co-participants, and frequency of activities between practitioners reporting and not reporting CP

The logistic regression model was statistically significant, X^2^ (8, *N* = 511) = 43.60, *p* < 0.001, while Hosmer and Lemeshow test identified strong model fit, X^2^ (7, *N* = 511) = 6.01, *p* = 0.539. The model explained 11.0% (Nagelkerke) of the variance in reporting of CP. PRCP were 113% less likely to be self-introduced (OR = 0.45, 95% CI [0.29, 0.70], *p* < 0.001) and 138% less likely to be introduced by a partner (OR = 0.42, 95% CI [0.26, 0.70], *p* < 0.001) to BDSM (Table 4). Reporting CP did not appear to influence who someone co-participated with. This reinforces the above finding that many of those reporting CP did not have difficulties finding co-participants. However, PRCP were 168% more likely to cite mental health benefits as a driving purpose (OR = 2.68, 95% CI [1.31, 5.49], *p* = 0.007) for their continued participation in BDSM.

**Figure 3.**
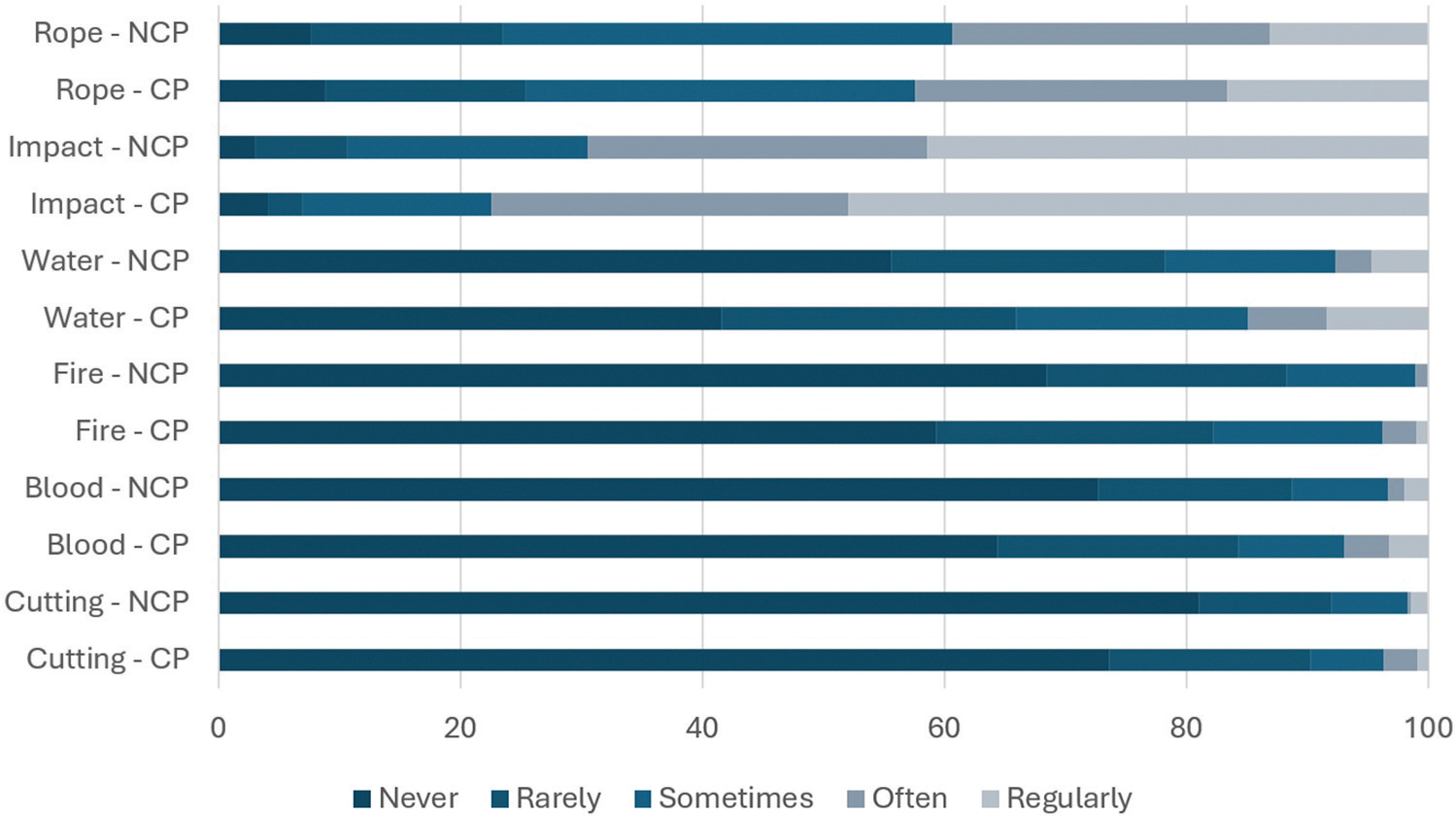
Frequency of participation in BDSM activities by practitioners reporting and not reporting chronic pain. *NCP* = practitioners who did not report chronic pain, CP = practitioners who reported experiencing chronic pain

Finally, the frequencies at which BDSM PRCP and not reporting CP engaged in certain types of BDSM activities are presented in Figure 3. Rope was equally as ‘often’ or ‘regularly’ participated in for both groups (42.4% of PRCP vs 39.2% of participants not reporting CP). The same pattern emerged for impact (77.4% vs 69.5%) and cutting (3.7% vs 1.6%). However, PRCP more ‘often’ or ‘regularly’ participated in fire (3.7% vs 1.0%), water (14.9% vs 7.7%), and blood (6.9% vs 3.3%) play. The effect sizes for water (0.16), fire (0.13), and blood (0.11) were small (Table 5).

## Discussion

Research is beginning to consider whether engagement in BDSM may lead to positive secondary outcomes in aspects of practitioners’ lives beyond sexual satisfaction (Thomas, 2020; Turley, 2024). Building upon the concept of unanticipated benefits, this is the first large-scale study to explore whether PRCP perceive challenges and/or benefits to participating in BDSM related to their experience of CP. In exploring this topic, four key findings emerged. First, many PRCP endorsed physical and mental benefits resulting from engagement in BDSM, while some reported little to no benefits. Second, experiences of CP can lead to challenges in getting wants and needs met, but these do not appear to deter their long-term participation. Third, PRCP are slightly over-represented within BDSM communities compared to the general population. Fourth, PRCP do not appear to seek out BDSM specifically for pain management. However, they are more likely to cite mental health as a reason for continued participation in BDSM and more frequently engage in some intense sensation BDSM activities.

BDSM appears to provide self-perceived benefits to many PRCP, including the experience of an endorphin rush. Within the kink community, the altered state of consciousness and euphoria resulting from an endorphin rush is known as ‘sub-space’ or ‘dom-space’, depending on a person’s role during the activity, and is generally considered a positive experience (Ambler et al., 2017). This finding suggests that there is potential for PRCP to be experiencing a form of pain offset relief from their participation in BDSM; however, further research specifically exploring this potential relationship, and its effectiveness in pain management for PRCP is needed.

Supporting findings from smaller studies, participants also cited benefits related to empowerment, reframing of limitations, and pain relief (K. Brown & Iverson, 2023; Sheppard, 2018). Building upon this prior work, participants were equally likely to cite additional mental benefits (i.e. engaging emotionally with pain and improving the ability to speak about pain) and physical benefits (i.e. strengthened pain tolerance). However, self-perceived benefits from participating in BDSM were not universal. Managing pain through negotiation, speaking about pain experienced, empowerment and strengthening pain tolerance were equally as likely to being reported as experienced ‘often’ or ‘regularly’ as ‘not at all’. This is unsurprising given the varying efficacy of treatment methods for those with CP (Urits et al., 2021).

Although CP did present certain challenges to getting one’s wants and needs met during BDSM activities, potentially the result of discomfort or additional pain, this did not appear to lead to community-level negative effects. PRCP did not feel excluded or have difficulties finding co-participants and appeared to participate with an equal variety of partners as those without CP. This may point to the generally welcoming and accepting nature of BDSM communities compared to other spaces (Graham et al., 2016). Likewise, any negative effects related to CP that arose from participating in BDSM did not seem to impact long-term participation as there was no difference in years of participation between those reporting and not reporting CP.

Despite reporting benefits to pain management from participating in BDSM, findings suggest that those with CP do not necessarily seek out BDSM as a form of treatment. PRCP were less likely to be self-introduced and their historical timelines of BDSM participation (e.g. age of first exposure) were like those without CP. This aligns with prior research on alternative treatment methods for CP, whereby people may not engage in something because they know it will improve pain management but rather come to find it beneficial once they begin participating (Holtrop et al., 2019). However, PRCP were more likely to cite ‘mental health’ as a reason for their continued engagement in BDSM. It is unclear whether this improved mental health is related to their CP, but there is a known negative relationship between mental health and CP (Tsang et al., 2008; Vadivelu et al., 2017). Therefore, it is possible that practitioners experienced improved mental health secondary to BDSM participation, in part due to an improved ability to manage their CP. This may fuel their continued participation in BDSM despite any challenges experienced because of their CP and warrants further study.

PRCP appear to account for a slightly higher proportion (41.9%) of BDSM practitioners than the 30% found in the general population (Cohen et al., 2021). Like the general population, women (Tsang et al., 2008) and people with lower educational levels (Dahlhamer et al., 2018) were more likely to report experiencing CP. However, it is important to acknowledge that a non-representative sample was used in the current study, and that participants self-identified as experiencing CP. This non-clinical definition and the non-representative sample (e.g. 62.4% were assigned female at birth) are likely account for this discrepancy. Therefore, additional representative research is needed to examine whether the prevalence of CP is truly higher in BDSM communities. Despite common challenges in obtaining racially and ethnically diverse samples within BDSM studies (A. Brown et al., 2020; Martinez, 2021), it is important that these populations within BDSM communities are analysed, given their triple marginalisation (CP, BDSM, and race/ethnicity) and their increased likelihood of experiencing CP (Overstreet et al., 2023; Zajacova et al., 2022).

While PRCP were no more likely to seek out intense sensation-*receiving* roles (e.g. submissive, masochist vs dominant, sadist), they were more likely to seek out engagement in ‘edge-play’ activities, namely water-, fire-, and blood-play. Although what constitutes edge-play is undefined, it is generally considered to include activities that are viewed as more intense or risky. Edge-play may include activities that involve higher risk of exposure to certain bodily fluids that can increase risk of infection (e.g. urine, blood). It may also include activities that have higher risk of harm to participants (e.g. accidental burns, unintentional wounds, unwanted permanent scars). It is unclear why these activities would be more common among BDSM PRCP, but one hypothesis is that those with CP seek out more intense sensory experiences because they are likely to provide a stronger euphoric rush, regardless of whether the experience is specifically pain-based or derived from increased risk. However, greater exploration of the reasons for participating in certain BDSM activities by practitioners with and without CP is needed.

### Limitations and directions for future research

The large sample size, diversity of benefits and challenges explored, and the self-perceived linkage of benefits to participation in BDSM by PRCP provides additional evidence of the intersection between these two populations. However, limitations in the design and depth of study questions make the interaction between CP and BDSM unclear. With this in mind, we outline three directions for future research to better clarify the connection between the two.

First, participants were recruited through online convenience sampling, which results in selection bias and may limit generalisability. However, the online nature of the survey allows for participation of practitioners who do not engage in BDSM publicly and therefore would not be attending in-person events. Participants were also not provided with a standardised definition of CP, and self-identified as experiencing CP. Additionally, no data was collected on the type and severity of CP reported. Combined, these make it difficult to fully characterise the experiences of PRCP, given the multitude of possible pain types and locations of CP (e.g. headache vs fibromyalgia). Therefore, research that uses clinical definitions of CP and inquires about specific diagnoses and treatment methods attempted would better parse out whether BDSM is beneficial for some types of CP or PRCP and not others, or whether it is more universal, thus increasing generalisability of findings.

Second, while we can argue that PRCP endorse benefits to their CP from BDSM participation, we were unable to determine to what degree engagement in BDSM activities is dictated by CP. As the survey was cross-sectional, we were also unable to determine causality or true significant benefits of BDSM for CP management. Future longitudinal research would be needed to assist in answering this question. This includes whether some BDSM activities are more beneficial or pose greater challenges for certain types of CP. For example, research examining activities such as electrical play, that make use of the same or similar tools and devices as those currently used in CP treatment (Johnson et al., 2022), would be an important avenue for further study. Additionally, reported euphoria may come from different aspects of BDSM practice there were not expounded upon in this study (e.g. the eroticisation of BDSM-related pain, overcoming said pain, ‘taking’ pain for a partner). Furthermore, once PRCP identify that some BDSM activities result in benefits to their CP, does this become a primary motivation for continued participation in BDSM or does it continue to be a secondary benefit? Finally, do PLWCP have higher pain thresholds and find that the benefits of BDSM participation diminish over time, necessitating more intense sensations more frequently (e.g. higher engagement in edge-play).

Third, we are unable to determine how pain during BDSM activities may be used to address CP. For example, whether the type of CP being experienced impacts the choice of BDSM activities (e.g. use of impact-play implements that provide sharp/sting versus dull/thud pain). Do BDSM activities result in similar pain to practitioners’ CP or do activities oppose it? This includes whether the administration of pain is focused on or avoids the body areas where CP manifests. Finally, for PRCP that are dominant, does their application of pain or intense sensation to their co-participant(s) mirror or differ from their own pain? Additional research is needed to answer these questions, and qualitative exploration could offer a more nuanced understanding of people’s experiences.

## Conclusions

As minimal research exists on the connection between BDSM and CP, study results make an important contribution to the existing foundation of knowledge on the subject. Findings suggest that many PRCP attribute some amount of short-term relief from CP by engaging in BDSM. Thus, the role of intense sensory experiences during BDSM activities in the experience and management of CP deserves further exploration. These findings also may provide additional support to the pain offset relief and gate control theories related to the role of peripheral nerve stimulation in chronic pain treatment. However, prior to drawing any firm conclusions on the use of BDSM activities as a complementary, non-pharmacological, CP treatment method, additional research is needed. This is of particular importance given various consent and safety risks inherent in BDSM activities, particularly those involving edge-play. That being said, BDSM communities may be interested to utilise this knowledge to better support community members living with CP by hosting workshops that address specific challenges and/or developing educational resources to promote safer and more supportive practices for practitioners living with CP. Nevertheless, this study provides further evidence that many of those who engage in BDSM obtain secondary benefits to their mental health and well-being beyond sexual-related benefits.

## Figures and Tables

**Table 1.** Demographic differences between BDSM practitioners reporting and not reporting chronic pain.

	All participants (*N* = 525)		Practitioners reporting chronic pain (n = 220^+^)		Practitioners without chronic pain (*n* = 305)	
			
	N	%	n	%	n	%

**Generation**	X^2^ (3, *N* = 517) = 4.88, *p* = 0.181				Cramer’s V = 0.097	

*Baby Boomer (’46–’64)*	69	13.3	36	16.6	33	11.0
*Generation X (’65–’80)*	139	26.9	62	28.6	77	25.7
*Millennial (’81–’96)*	235	45.5	90	41.5	145	48.3
*Generation Z (’97–’10)*	74	14.3	29	13.4	45	15.0

**Sex Assigned at Birth**	X^2^ (1, *N* = 515) = 8.82, *p* = 0.003				Phi = 0.131	

*Male*	192	37.1	63	32.8	129	46.1
*Female*	323	62.4	149	67.2	174	53.9
*Intersex*^++^	3	0.6	3	0.0	0	0.0

**Transgender**	X^2^ (1, *N* = 520) = 0.465, *p* = 0.495				Phi = 0.030	

*Yes*	52	10.0	24	11.1	28	9.2
*No*	468	90.0	193	88.9	275	90.8

**Gender**	X^2^ (2, *N* = 521) = 10.145, *p* = 0.006				Cramer’s V = 0.140	

*Man*	175	33.6	56	25.8	119	39.1
*Woman*	264	50.7	122	56.2	142	46.7
*Gender Diverse*	82	15.7	39	18.0	43	14.1

**Sexual Orientation**	X^2^ (3, *N* = 519) = 1.54, *p* = 0.673				Cramer’s V = 0.054	

*Heterosexual*	197	38.0	77	35.5	120	39.7
*Bisexual*	164	31.6	68	31.3	96	31.8
*Pansexual*	76	14.6	35	16.1	41	13.6
*Another SO*	82	15.8	37	17.1	45	14.9

**Race**	X^2^ (1, *N* = 518) = 0.052, *p* = 0.819				Phi = 0.010	

*White*	437	84.4	184	84.8	253	84.1
*Persons of Color*	81	15.6	33	15.2	48	15.9

**Country**	X^2^ (3, *N* = 517) = 13.962, *p* = 0.003				Cramer’s V = 0.164	

*U.S.A.*	321	62.1	150	68.8	171	57.2
*Canada*	73	14.1	34	15.6	39	13.0
*UK*	40	7.7	11	5.0	29	9.7
*Elsewhere*	83	16.1	23	10.6	60	20.1

**Education Level**	X^2^ (3, *N* = 522) = 11.931, *p* = 0.008				Cramer’s V = 0.151	

*HS or lower*	45	8.6	20	9.2	25	8.2
*Some College*	147	28.2	74	33.9	73	24.0
*Bachelor’s Degree*	192	36.8	82	37.6	110	36.2
*Graduate Degree*	138	26.4	42	19.3	96	31.6

**Occupation Status**	X^2^ (3, *N* = 500) = 7.914, *p* = 0.048				Cramer’s V = 0.126	

*Employed*	394	75.0	154	73.0	240	83.0
*Student*	36	7.2	21	10.0	15	5.2
*Retired*	34	6.8	18	8.5	16	5.5
*Unemployed*	36	6.9	18	8.5	18	6.2

**Relationship Status**	X^2^ (3, *N* = 514) = 1.488, *p* = 0.685				Cramer’s V = 0.054	

*Married*	146	28.4	60	28.4	86	28.7
*Dating*	133	25.9	61	28.5	72	24.0
*Single*	124	24.1	48	22.4	76	25.3
*Other*	111	21.6	45	21.0	66	22.0

+: combines ‘yes’ with ‘yes, but I want to skip the questions about chronic pain’.

++: analysis excludes intersex participants.

**Table 2.** Differences in BDSM roles between practitioners reporting and not reporting chronic pain.

	All participants (*N* = 525)		Practitioners reporting chronic pain (*n* = 220^+^)		Practitioners without chronic pain (*n* = 305)	
			
	N	%	n	%	n	%

**BDSM Role**	X^2^ (2, *N* = 499) = 0.014, *p* = 0.993				Cramer’s V = 0.005	

*Dominant roles*	108	21.6	45	21.4	63	21.8
*Submissive roles*	241	48.3	102	48.3	139	48.1
*Both (e.g. Switch)*	150	30.1	63	30.1	87	30.1

**Sadism**	X^2^ (1, *N* = 519) = 0.904, *p* = 0.342				Phi = 0.042	

*Sadist*	76	14.6	28	12.9	48	15.9
*Non-Sadists*	443	85.4	189	87.1	254	84.1

**Masochism**	X^2^ (1, *N* = 519) = 3.715, *p* = 0.054				Phi = 0.085	

*Masochist*	97	18.7	49	22.6	48	15.9
*Non-Masochists*	422	81.3	168	77.4	254	84.1

+: combines ‘yes’ with ‘yes, but I want to skip the questions about chronic pain’.

**Table 3.** Relationship between reporting chronic pain and age of participation pathways into BDSM.

	Practitioners reporting chronic pain	Practitioners without chronic pain	Significance		
	
Average Age	Mean (SD)	Mean (SD)	t-value (df)	P value	Cohen’s d

First Learned About BDSM	18.50 (9.26)	18.29 (8.56)	.274 (511)	.784	.026
First Interested in BDSM	20.78 (10.01)	20.45 (9.73)	.378 (517)	.705	.034
First Participated Privately	24.74 (11.33)	24.05 (10.28)	.714 (515)	.476	.064
First Participated Publicly	29.47 (12.31)	30.39 (9.80)	.712 (251.88)	.477	.084
Years of Participation	16.77 (13.22)	15.04 (11.90)	1.558 (512)	.120	.139

**Table 4.** Relationship between source of introduction, co-participants, and reason for participation for practitioners reporting and not reporting chronic pain.

	Practitioners reporting chronic pain	Practitioners without chronic pain	EXP(B)	95% CI

Self-Introduction	118 (36.4%)	206 (63.6%)	0.45**	0.29–0.70
Friend Introduction	44 (60.3%)	29 (39.7%)	1.69	0.97–2.95
Partner Introduction	36 (31.3%)	79 (68.7%)	0.42**	0.26–0.70
Participate with Friends	85 (49.4%)	87 (50.6%)	1.44	0.96–2.18
Participate with Strangers	59 (45.4%)	71 (54.6%)	1.11	0.71–1.74
Participate Alone	56 (42.7%)	75 (57.3%)	0.94	0.60–1.47
Participate with Sex/Romantic Partner(s)	188 (41.7%)	263 (58.3%)	0.91	0.53–1.57
Reason for Participation – Mental Health	25 (65.8%)	13 (34.2%)	2.68*	1.31–5.49

* *p* < 0.01

** *p* < 0.001.

**Table 5.** Linear-by-linear association analyses for six BDSM activities between practitioners reporting and not reporting chronic pain.

Activity (sample size)	Linear-by-linear assoc. (df)	Significance	Effect size (Cramer’s V)

Rope (*n* = 518)	0.15 (1)	0.69	0.066
Impact (*n* = 519)	3.13 (1)	0.08	0.127
Water (*n* = 511)	12.21 (1)	<0.01	0.160
Fire (*n* = 515)	6.95 (1)	<0.01	0.128
Blood (*n* = 516)	4.51 (1)	0.03	0.110
Cutting (*n* = 516)	2.53 (1)	0.11	0.137

## Data Availability

Available upon request.
